# Neuroimmune crosstalk in the central nervous system and its significance for neurological diseases

**DOI:** 10.1186/1742-2094-9-155

**Published:** 2012-07-02

**Authors:** Li Tian, Li Ma, Tiina Kaarela, Zhilin Li

**Affiliations:** 1 Neuroscience Center, Viikinkaari 4, FIN-00014, University of Helsinki, Helsinki, Finland

**Keywords:** Microglia, Astrocyte, Neuron, Neuroinflammation, Innate immunity, Adaptive immunity

## Abstract

The central nervous system (CNS) is now known to actively communicate with the immune system to control immune responses both centrally and peripherally. Within the CNS, while studies on glial cells, especially microglia, have highlighted the importance of this cell type in innate immune responses of the CNS, the immune regulatory functions of other cell types, especially neurons, are largely unknown. How neuroimmune cross-talk is homeostatically maintained in neurodevelopment and adult plasticity is even more elusive. Inspiringly, accumulating evidence suggests that neurons may also actively participate in immune responses by controlling glial cells and infiltrated T cells. The potential clinical application of this knowledge warrants a deeper understanding of the mutual interactions between neurons and other types of cells during neurological and immunological processes within the CNS, which will help advance diagnosis, prevention, and intervention of various neurological diseases. The aim of this review is to address the immune function of both glial cells and neurons, and the roles they play in regulating inflammatory processes and maintaining homeostasis of the CNS.

## Introduction

Our understanding on the reciprocal relationship between the nervous and immune systems has developed rapidly within the past decade. Inflammatory response has been associated with pathological processes in a wide range of brain disorders, including not only the conventional inflammatory conditions in autoimmune diseases, traumatic brain injuries, stroke, and neurodegenerative diseases [1,2], but more importantly also neurodevelopmental defects in schizophrenia, epilepsy, and autism [3-5]. However, immune responses are specialized within the brain and differ considerably from those in the periphery. Such differences endow the central nervous system (CNS) with an immune-privilege status, and provide challenges for therapeutic interventions to various CNS diseases [6-8]. Hence, a thorough insight into the immunological processes within the CNS and their involvement in neurodevelopment and neuropathology is pivotal for current researchers. This article aims at providing readers the updated knowledge of the immune regulatory functions of the CNS residential cells: glia and neurons. Since immune functions of glia are much better known than those of neurons [4,5,9], we will emphasize more on the latter ones in this review. We posit that neurons, glia, and peripheral immune cells form an integrative network to actively regulate immunological processes that affect brain functions, which will be dissected in detail here.

### Glia as innate immune cells in the CNS

Although the CNS is relatively secluded from the peripheral immune system, it has its own residential immune network, in which glial cells (mainly microglia and astrocytes) not only serve supportive and nutritive roles for neurons, but also defend the CNS from stress and pathogenic insults by transiently up-regulating inflammatory processes [9]. These processes are usually kept in check by other endogenous anti-inflammatory and neuroprotective responses that return the CNS back into homeostasis. However, excessive or prolonged glial activation results in a more severe and chronic neuronal damage that eventually propagates neuroinflammation and neurodegeneration, suggesting the delicacy of tipping the balance between neuroprotection and neurotoxicity [10]. This is substantiated by studies showing that similar to chronic neurodegenerative diseases, microglia in the aged, but otherwise healthy brain are in a more reactive state compared with the younger cohorts. These already ‘primed’ microglia release excessive amounts of pro-inflammatory cytokines upon stimulation, which subsequently exaggerates degenerative changes [11,12].

**Figure 1:**
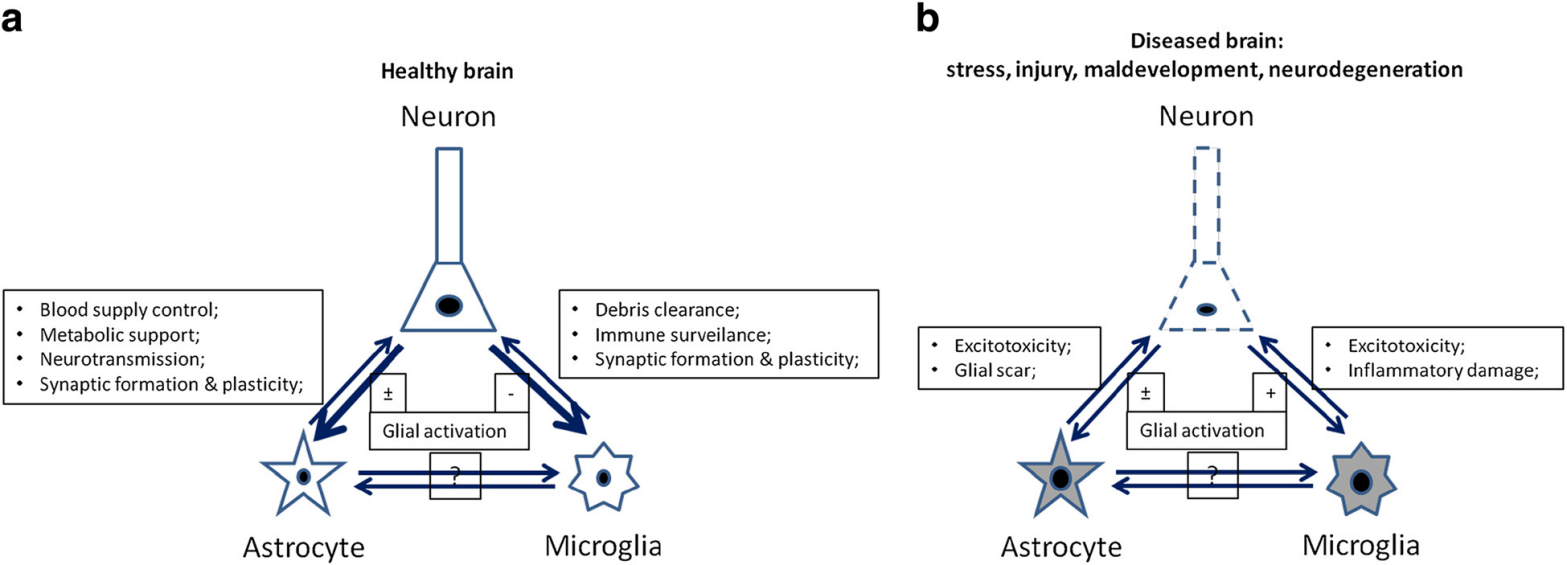
**A simplified schematic illustration of the interaction between the trios of neurons, astrocytes, and microglia in the CNS under the normal (a) and pathological conditions (b).** Healthy neurons are able to tightly regulate the activation of their neighboring glial cells. Meanwhile both astrocytes and microglia help maintain the neuronal activity. Under various diseased conditions, this homeostasis is broken so that neurons lose their controlling ability but instead deliver damage signals to glial cells, which in turn may exacerbate neuronal damage through inflammation. The cross-talk among astrocytes and microglia themselves and its aftermath on neurons is currently not very clear.

It is interesting to note that glial cells are not only able to directly detect invaded pathogens in the CNS, but can also sense the immune responses occurred in the periphery through cytokines and chemokines, which alert the CNS through humoral and/or neurochemical pathways. Glia in turn elicit a broad spectrum of chemokines and cytokines themselves, which cause or exaggerate the so-called sickness behaviors or the analogous depressive symptoms [13,14], indicating an active communication of the central glial cells with the peripheral immune system. Under homeostatic conditions, the peripherally and centrally synthesized inflammatory proteins play key roles in orchestrating the adaptive reactions of the CNS towards physical or emotional stresses [13,14]. Yet again, they can be harmful for neuronal development and plasticity when the immunological process turns more chronic or in those individuals who are predisposed to certain neurological diseases, such as schizophrenia, epilepsy, and autism, and therefore do not tolerate the immune stimulations as well as the healthy people (Figure 1) [5,11,13,15].

### Microglia

Microglia comprises 5% to 20% of all glial cells in various brain regions. As commonly known, they are the major phagocytic cells that provide the first line of defence for the CNS [16]. In respect to neuroimmune cross-talk, microglia play the most direct and perhaps also the most important role in sensing and modulating neuronal activities. Resting microglia acquire a ramified but nevertheless active morphology normally, with minimal expression of myeloid-monocytic markers such as Fc receptors-cluster of differentiation (CD) 32 and CD64, complement receptors (CR)-3 and −4, (also named as CD11b and CD11c integrins, respectively), major histocompatibility complex (MHC) class I and II, and CD45 [17] (Table 1). Once challenged by inflammation, microglia become rapidly ameboid and up-regulate a variety of cell surface receptors involved in innate immune responses. These receptors include pattern recognition receptors (PRR), such as toll-like receptors (TLR) and receptors for advanced glycation end products (RAGE) and scavenger receptors (CD36, CD91), as well as phagocytic receptors, such as CR-3, -4, and triggering receptor expressed on myeloid cells (TREM) (Tables 1 and 2) [17,18].

**Table 1:** Immune properties of glia and neurons in the CNS

**Properties**	**Microglia**	**Astrocyte**	**Neuron**
*Innate immunity*			
PRRs	High [16-19]	High [20]	Low [21]
Phagocytic receptors	High [16-19]	Low [20]	Unknown
Cytokine production			
Pro-inflammatory	Yes [16-19]	Yes [10,20,22]	Yes [8,23]
Anti-inflammatory	Yes [16-19]	Yes [10,20,22]	Yes [23-28]
*Adaptive immunity*			
MHC classes	I & II [17,29] Inducible	I & II [22,29-31] Low/inducible	I [32] Inducible
Co-stimulatory molecules	Inducible [17,29]	Low/inducible	Unknown
Antigen presentation	Yes [17,29]	Controversial [29]	Unknown
T-cell differentiation	Th1, Th2 [17,29,33]	Th2 [33,34],	Largely unknown
		Th1 [33,34],	
		Treg [35,36]	
*Induction of apoptosis*			
T cells	Yes [18,29,37-41]	Yes [18,29,37-41]	Yes [18,29,37-41]
Microglia	Yes [18,29,37-41] (self-limiting)	Yes [18,29,37-41]	Yes [18,29,37-41]

**Table 2:** Pro- and anti-inflammatory molecules expressed by glia and neurons

**Properties**	**Promotion of inflammation**	**Inhibition of inflammation**
*Microglia*		
Soluble factors	TNF, IFN-γ, IL-1β [16-19];	IL-4, IL-10, IFN-β, TGF-β [16-19];
	CXCL1,2,12, CCL2,5,10,19 [42];	BDNF, GDNF [42];
	Glutamate; NO, ATP [16-19]	TIMPs [43]
	MMPs [43]; Complements [18,44];	
	HMGB1, heat-shock proteins [25,45-47]	
Membrane proteins	TLRs, RAGE, LFA-1, MAC-1, CRs, FcRβ [16-19,21,25,45-47]	CD45, CD91, CD200R, CD172a [18,48]; CX_3_CR1 [49]; TREM-2 [50]; FasL, Fas [38,39]
*Astrocytes*		
Soluble factors	TNF, IFN-γ, IL-1β [10,20,22] CXCL1,2,12, CCL2,5,10,19 [20,42];	IL-4, IL-10, IFN-β, TGF-β [10,20,22] Proteoglycans [51,52];
	Glutamate; NO, ATP; MMPs [10,20,22]; Complements [18,20,44] HMGB1, heat-shock proteins [25,45-47]	BDNF, GDNF [42,53,54]; TIMPs [43]
Membrane proteins	TLRs, RAGE, ICAM-1, CRs [21,25,46]	FasL [37,38], Complement inhibitors [20]
*Neurons*		
Soluble factors	CXCL10, CCL21 [8,23,42];	TGF-β [27,28]; CX_3_CL1 [49,55];
	Glutamate, dopamine [23];	GABA [56,57]; VIP [58,59]; NE
	NO, ATP; Substance P [23]; MMPs [43]; HMGB1, heat-shock proteins [25,45-47]	[60,61];
		Proteoglycans [51,52];
		NGF, BDNF, NT3, GDNF, CNTF [42,53,54]
Membrane proteins	TLRs [21]	CD22 [62], CD47 [63,64], CD200 [48], ICAM-5 [65,66], FasL [37,38]

Phagocytosis is an important mechanism for microglia to control neuronal apoptosis. When apoptosis of neurons occurs, for example, at the early developmental stage, or due to neurodegeneration, clearance of apoptotic cell debris in time through phagocytosis is vital for the reminiscent neurons to avoid collateral inflammation-induced damage [19,67]. Apoptotic cells express cell surface molecular patterns that act as ‘eat me’ signals. These signals are recognized by microglial PRRs, which rapidly initiate clearing processes [18]. On the other hand, insufficient phagocytic clearance of cell debris following neuronal injury is an important pathogenetic factor in propagation of neurodegeneration, as illustrated by numerous studies on Alzheimer’s disease (AD) [16,19].

Besides controlling neuronal apoptosis, microglia secrete a wide variety of cytokines, complements, and growth factors that have been implicated in regulating synaptic formation and plasticity [67]. With the help of the complement system, unwanted synapses are efficiently removed by phagocytosis under both developmental and pathological conditions [68]. Mice deficient in complement C1q or C3 exhibited sustained defects in elimination of the CNS synapses [44]. More importantly, the orthodox view that microglial activity is only relevant for the pathological processes of neuronal pruning has been overturned by the recent evidences showing that they are highly sensitive for the neighboring neuronal activities and control dendritic spine density under physiological conditions [69,70]. Furthermore, a recent elegant work by Derecki *et al.* demonstrated that microglia from bone marrow of wild-type mice attenuated Rett syndrome, an X-linked autism spectrum disorder [71]. Undoubtedly, emerging evidences with the help of modern imaging and analytical technologies will shed light into the physiological functions of microglia in the coming years.

Another important function of microglia is the presentation of foreign antigens to T lymphocytes. In the normal CNS, antigen presenting cells (APC) are mainly confined to dendritic cells and macrophages located in the meninges, choroid plexuses, and perivascular spaces [29]. MHC class I and II molecules are expressed at low levels by microglia, as they are actively down-regulated by the immune-quiescent microenvironment of the CNS. However, upon activation, these molecules are up-regulated together with the co-stimulatory receptors CD40, CD80 (B7-1), CD86 (B7-2), and leukocyte function-associated antigen-1 (LFA-1, CD11a/CD18 integrin), which subsequently induces optimal APC functions and T-cell activation (Tables 1 and 2) [17,29].

Unarguably, microglial cells have to be kept in check under normal conditions. Furthermore, as an excessive inflammation causes disastrous bystander damage to the CNS, activation of microglia under pathophysiological conditions of stress or mild malaise has to be restricted by counter-regulatory mechanisms to resurrect the CNS homeostasis (Figure 1) [16]. Unfortunately, how the balance is achieved and how to beneficially modulate it in psychological and neurological diseases are still unclear so far.

### Astrocyte

Astrocytes have been traditionally viewed as supportive cells for neurons, which are responsible for the CNS homeostasis and neuronal functions [72] (Figure 1a). Their functions as innate immune cells are somehow less appreciated as compared to microglia. Nevertheless, astrocytes have been known to form the glia limitans around blood vessels, thereby restricting the entry of immune cells through the blood-brain barrier (BBB) into the CNS parenchyma [73]. Emerging evidences have highlighted the importance of this cell population in the regulation of local innate and adaptive immune responses [20].

Astrocytes express a variety of PRRs involved in innate immunity, including TLRs, scavenger receptors, mannose receptors, and CRs (Tables 1 and 2) [20]. Following PRR engagement, astrocytes secrete cytokines, chemokines, and neurotrophins that target neighboring glial cells and neurons [10,20]. Released cytokines also promote the leakage of BBB, resulting in the recruitment of immune cells from the blood circulation into the CNS parenchyma. These altogether amplify both the initial innate immune responses and the upcoming adaptive immune responses, which can result in the elimination of infectious or injurious insults and restoration of tissue integrity or scar formation [74].

Astrocytes are active players in the AD and multiple sclerosis (MS) [22,75]. Under pathological conditions, astrocytes undergo a series of structural and functional changes collectively referred to as astrogliosis. Amyloid β-peptide (A-β) plays an important role in astrocyte stimulation [76]. Accumulation of astrocytes around the senile plaques and neurofibrillary tangles is one of the hallmarks of the AD [75]. Upon A-β stimulation, astrocytes secrete various chemokines that recruit microglia and monocyte/macrophages to the plaques with the concomitant release of neurotoxins and pro-inflammatory cytokines that contribute in concert to neurodegeneration [75]. Astroctyes are also involved in the MS disease through release of pro-inflammatory cytokines, matrix metalloproteinases (MMP), and free radicals that result in recruitment of autoimmune cells, oligodendrocyte destruction, and demyelination (Table 2) [22].

Another interesting, yet controversial, property of astrocytes is their antigen-presenting ability. *In vitro* studies have shown that astrocytes express low levels of MHC and co-stimulatory molecules upon stimulation by cytokines, and are capable of processing and presenting myelin protein epitopes to T cells, inferring that they can also be APCs [30,31]. However, given the more efficient antigen presentation capabilities of microglia and other infiltrating APCs in the CNS, the *in vivo* significance of this function of astrocytes is unclear (Table 1) [9,22,29].

Despite of these controversies, the ability of astrocytes to regulate the antigen-presenting activities of other APCs and T-cell activation has been clearly established. The fact that the expression levels of MHC and co-stimulatory molecules are low even after stimulation *in vitro* suggests that astrocytes may be prone to induce T helper (Th) 2 rather than Th1 responses. Indeed, earlier studies showed that while microglial cells seem to be efficient in activating both Th1 and Th2 cells, astrocytes stimulate only Th2 cells [33]. Microglia and astrocytes also produce chemokines that differentially affect the recruitment of Th1 and Th2 cells (Tables 1 and 2) [34].

Additionally, astrocytes have been shown to induce anergy of Th cells via cytotoxic T-lymphocyte antigen 4 (CTLA-4, CD152) [77] or promote recruitment and proliferation of regulatory T cells (Treg) via the anti-inflammatory cytokine transforming growth factor β (TGF-β) [35] and chemokine CXCL12 (stromal cell-derived factor-1, SDF-1) [36]. Astrocytes can also secrete other anti-inflammatory cytokines or soluble factors, including interleukin (IL)-10, interferon (IFN)-β, and neurotrophins, which are able to suppress T cell and microglial activation (Table 2) [10,20,22]. So it seems that a complex network of interactions between neurons, astrocytes, microglia, and T cells is involved in determining the balance of pro- *vs.* anti-inflammatory signals, which in turn affect the outcome of immune responses within the CNS.

Collectively, the above information indicates that both microglia and astrocytes play an active and dual role in the CNS inflammatory diseases. They not only have the ability to enhance immune responses and promote neurodegeneration, but can also be protective and limit the CNS inflammation.

### Neurons actively regulate innate and adaptive immune responses in the CNS

While a plethora of data has highlighted the immune-regulatory functions of microglia and astrocytes, the roles that neurons play in this arena are under-toned [8,23]. Neurons have primarily been regarded as victims of immune attack, and their participation in the CNS immune responses as passive.

Nevertheless, the CNS has been known to regulate peripheral innate immune responses through hormonal and neuronal circuits [24,25]. The neuroendocrine-mediated stress responses induced by infection or traumatic injuries generally inhibit innate immune responses at the systemic level [24]. The sympathetic and parasympathetic nervous systems also sense injury and infection at the regional level, and reflectively modulate immune responses through the adrenergic and cholinergic anti-inflammatory pathways [24,25]. It is noteworthy that the major neuroendocrine hormones and neurotransmitters that control the peripheral immunity also play important roles in controlling the functions of various CNS cells, not only in response to immune stimuli, but also to stress and emotional arousal, in order for an organism to adapt to its environment. Therefore the central roles they play in regulating immune responses of the CNS should not be overlooked [26].

Reciprocally, besides behaving as sentinels to fend off pathogens, both central and peripheral immune cells participate in neurodevelopment and cognitive functions of the brain as well. This is substantiated by the recent evidences that microglia intimately interact with neurons to monitor their activities, as previously discussed. Additionally, peripheral adaptive immune cells may also regulate neurogenesis, learning, and emotional behaviors of animals [78]. Deficiency in the adaptive immunity of severe combined immunodeficiency (SCID) or recombination activating gene (RAG)-1(−/−) mice is associated with reduced neurogenesis and impaired learning and memory [79,80]. Systemic depletion of CD4+ T cells led to significantly reduced hippocampal neurogenesis and impaired reversal learning in the Morris water maze, and repopulation of RAG-2(−/−) mice with CD4+ T cells increased neural precursor cell proliferation [81]. Recently, immune activity has also been found associated with programming of the hypothalamus-dependent stress axis. Germ-free mice, which have the ill-balanced immune system, were shown to display increased motor activity and reduced anxiety as compared to mice with a normal gut microbiota [82]. Furthermore, repeated stress increased blood pressure in wild type but not RAG-1(−/−) mice, and adoptive transfer of T cells to RAG-1(−/−) mice restored blood pressure elevation in response to stress [83].

Bearing in mind that the CNS contains BBB that normally prevents immune cells from penetration [6,7], as often quoted by some hard-cored neuroscientists, it remains as a question as how the peripheral immune cells keep the communication with the CNS cells. One possibility is a direct humoral route mediated by circulating cytokines and/or chemokines since cytokines and their receptors are expressed by glial and neuronal cells in the adult CNS and are important mediators of various brain and behavioral functions [84]. Alternatively, innervation by the autonomic nerve fibers provides a reflective loop between the brain and the peripheral blood vessels and gut epithelia [85]. Although mechanisms for many of the above-described phenomena have not been well-characterized from a holistic level so far, it should not be ignored that accumulating clinical evidences have strongly suggested the correlation of narcolepsy, depression, irritable bowel syndrome, autism, and schizophrenia with immune activation [4,5,14].

One can envisage that the regulation of such immune activity is crucial as too little or too much can both lead to impaired neurogenesis and cognitive functions. Since neurons in the brain are very sensitive to changes in their surrounding milieu, and are poor to regenerate once damaged, a number of mechanisms exit to limit the immune-mediated neurotoxicity and the collateral tissue damage, which were collectively denoted as immune privilege [6,7]. Here, we recount the current advances in this research field and summarize the mechanisms on neuronal regulation of innate and adaptive immunities. We believe that the CNS actively interacts with the immune system and provides several specific mechanisms to regulate the central immune responses, which involves not only microglia and astrocytes, but also neurons themselves (Tables 1 and 2; Figure 1a) [8,23].

### Soluble factors and their receptors

Neurons may inactivate T cells and microglia by both contact-dependent and -independent mechanisms [8]. Given that peripheral immune cells do not normally penetrate into the brain parenchyma, soluble factors provide an important means for the neuroimmune cross-talk. There are a number of neuronal soluble factors that potentially attenuate T- and microglial cell activation. These include anti-inflammatory cytokines [86], chemokines [42], neuropeptides [58], neurotrophins [42], and neurotransmitters [23] (Table 2).

TGF-β is the major anti-inflammatory cytokine needed for an organism to nurture Treg cells and keep autoimmune T cells at bay under the steady state. In the CNS, it is constitutively expressed in neurons [27,86]. Its importance in down-regulating microglial and autoimmune T-cell responses is substantiated by increased microglial activity and neuronal loss in the brains of TGF-β-deficient mice [28]. Additionally, fractalkine (CX_3_CL1, neurotactin) is the predominant chemokine expressed and released by neurons in the CNS, whereas its receptor CX_3_CR1 is primarily expressed in microglia. Fractalkine and its receptor play an important role in mediating interaction between neurons and microglia [55], which, on the one hand, controls inflammatory neurotoxicity in the brain as CX_3_CR1-deficient mice showed a massive activation of microglial cells upon repeated lipopolysaccharide (LPS) injection [49], and on the other hand, regulates the neuronal plasticity as the dendritic spines were increased in CX_3_CR1-deficient mice due to the inefficient synaptic pruning [70]. Furthermore, stressed neurons also secret semaphorin-3A and other substances to induce the apoptosis of activated microglia [87-89].

Neurotrophins are another group of soluble factors used by neurons to control immune cell functions. They play critical roles in neuronal survival, migration, and differentiation, and are potential drugs for the treatment of neurodegenerative diseases [90]. One aspect of their multiple effects on neurons is dampening inflammation-induced damage in the CNS. Among them, nerve growth factor (NGF) and brain-derived neurotrophic factor (BDNF) were investigated. NGF influences B-cell and T-cell functions and regulates macrophage migration into inflamed lesions outside of the nervous system [91]. Within the CNS, NGF was previously shown to inhibit MHC class II expression in microglia [53]. And BDNF was demonstrated to down-regulate the co-stimulatory molecules B7 and CD40 expression in microglia [54].

Additionally, neurons are equipped with various immunosuppressive neuropeptides and neurotransmitters, including vasoactive intestinal peptide (VIP), norepinephrine (NE), and γ-aminobutyric acid (GABA). VIP is a widely distributed neuropeptide with neuroprotective properties by inhibition of proinflammatory mediators, such as IL-6, tumor necrosis factor (TNFα), IL-12, and nitric oxide (NO), *in vivo* in various murine models of Parkinson’s disease, acute brain trauma, neuroinflammation, and cerebral ischemia [59]. NE is a catecholamine with roles both as a hormone and as a neurotransmitter that dampens cellular immunity systemically and suppresses neuroinflammation in the brain [60]. LPS-induced TNFα production in hippocampus is inversely correlated with the release of NE in an animal model of depression [61]. Furthermore, GABA, a main inhibitory neurotransmitter in the brain, has been shown to attenuate LPS-induced IL-6 and IL-10 production in microglia [57], and affect the entry of pathogenic T lymphocytes into the brain and T-cell proliferation [56].

### Cellular interactions

Neuronal membrane glycoproteins, such as CD22 [62,92], CD47 [63,64], CD200 [48,93], and neural cell adhesion molecule (NCAM) [94,95] have been shown to prevent microglial activation through interaction with their respective counter-receptors (Table 2).

The immunoglobulin superfamily (IgSF) member CD200 (OX2) is broadly expressed in neurons, endothelial, and immune cells, while its receptor, CD200R, which is also an IgSF molecule, is expressed predominantly by cells of the myeloid lineage, including microglia [48] and T cells [96]. Knocking out CD200 in mice results in a spontaneous microglial activation [48]. Similarly, antibody-mediated blocking of CD200R also leads to an aggravated clinical course of experimental autoimmune encephalomyelitis (EAE), accompanied by increased infiltration of T cells and macrophages [48]. Blocking CD200R on macrophages *in vitro* leads to enhanced IFN-γ-induced release of IL-6 and neuronal cell death in co-cultures with hippocampal neurons expressing CD200 [93].

Another IgSF and sialic acid-binding molecule, CD22, has been implicated in attenuation of the CNS immune responses as well. CD22 is expressed in cultured cortical neurons, and might mediate the binding of neurons to microglia through CD45 [62]. Ligation of microglial CD45 by CD22 has been shown to prevent the LPS-induced microglial production of the pro-inflammatory cytokine TNF [62,92].

Furthermore, the integrin-associated protein CD47 may also be important in down-regulating immune responses in the CNS. CD47 has been identified as a cellular ligand for the signal regulatory protein-α (SIRPα CD172a, which is also an IgSF member. Studies on SIRPα-deficient mice reveal that SIRPα is a negative regulator of macrophage phagocytosis [63], and ligation of monocyte SIRPα by CD47 down-regulates their TNF production [64].

Additionally, when mixed with glial cells *in vitro*, neurons inhibit the LPS-stimulated production of NO and TNF by glial cells. This effect is partially mediated through the IgSF adhesion molecule NCAM [94,95]. Chronic stimulation and interaction with apoptotic neurons induce microglial cells to release neuroprotective agents while inhibiting the production of NO and pro-inflammatory cytokines [1]. Furthermore, the receptor TREM-2 on microglia was shown to mediate the phagocytosis of apoptotic neurons while decreasing microglial pro-inflammatory responses, although its neuronal ligand has not been identified yet [50,97].

### Other mechanisms

Although experimental evidences supporting a direct interaction of neurons with T cells to prevent their antigen-dependent and -independent activation are currently still scant, this does not exclude the possibility that the T-cell-bearing counter-receptors for the above-mentioned neuronal molecules may regulate the amplitude of T cell receptor (TCR)-mediated activation of T cells [8]. We have previously shown that the neuronal intercellular adhesion molecule-5 (ICAM-5), an integrin ligand that regulates dendritic filopodia elongation and spine plasticity [65,98], down-regulates T-cell activation through interfering with the co-stimulatory function of integrin LFA-1 [66] (Table 2). In addition, semaphorins and their receptors plexins, in concert with neuropilin, have also been shown to attenuate T-cell activation and mitigate EAE [89,99-102]. Alternatively, neurons have been shown to convert T cells into Treg cells irrespective of their antigen specificity, which in turn suppresses EAE (Tables 1 and 2) [103,104]. And as suggested in the previous section, T-cell derived CD200R may also be important in dampening autoimmune responses in EAE [48].

Furthermore, neurons, microglia, and astrocytes have been shown to up-regulate Fas ligand (FasL, CD95L) under inflammatory conditions, which can induce apoptosis of activated T or microglial cells through the FasL-Fas pathway [37,38]. Administration of anti-FasL antibody to Lewis rat with EAE in the clinically recovery phase has been shown to reduce T-cell apoptosis, increase accumulation of T cells and macrophages at the site of inflammation, and delay spontaneous recovery of the animals [39]. Both T and microglial cells are more sensitive to Fas-dependent cell apoptosis than neurons and astrocytes [40,41], indicating the robustness of the later two cell types in limiting inflammatory insults through this pathway (Table 2).

It should be noted that the ability of neurons to limit immune responses in the CNS is largely dependent on their own integrity. At the early stage of pathological conditions, self-limiting machineries, such as apoptotic destruction of activated T cells, microglial cells, and injured neurons, may initially be able to keep the balance of protection *vs.* damage [18]. Once this is compromised, the inflammatory processes will be accelerated by neurons which turn on the expression of multiple pro-inflammatory cytokines and neurotoxic proteins such as A-β peptide, heat-shock proteins, and high mobility group box 1 (HMGB1, amphoterin) (Table 2; Figure 1b) [25,45]. These endogenous ‘dangerous signals’, also termed as damage-associated molecular pattern (DAMP) molecules, then agitate the already activated glial cells, and hence probably lead to their over-activation, which eventually exacerbates neuronal damages (Table 2) [46,47].

## Conclusions

Neurons, glial and immune cells form a coordinated network to maintain the homeostasis and restrict neuroinflammation in the CNS. This integrative network is not only involved in the pathogenesis of neuroinflammation, but more importantly, may also play a major role in the normal brain functions. The particular impact of glial and immune cells on the physiological *vs.* pathological process in the CNS is dependent on a number of factors that influence their state of activation. Here, we have summarized that neurons, the major effector cells for cognitive and motor function of an organism, provide environmental milieu that regulate glial and immune cell activation. Since inflammation is a double-edged sword for the CNS, improved knowledge on both beneficial and detrimental factors provided by neurons for the progress of inflammation should help develop better ways to treat various neurological diseases.

## Abbreviations

A-β, Amyloid β; AD, Alzheimer’s disease; APC, Antigen presenting cell; ATP, Adenosine tri-phosphate; BBB, Blood–brain barrier; BDNF, Brain-derived neurotrophic factor; CD, Cluster of differentiation; CR, Complement receptor; CNS, Central nervous system; CNTF, Ciliary neurotrophic factor; CTLA-4, Cytotoxic T-lymphocyte antigen 4; DAMP, Damage-associated molecular pattern; EAE, Experimental autoimmune encephalomyelitis; FasL, Fas ligand; GABA, γ-aminobutyric acid; GDNF, Glial cell line-derived neurotrophic factor; HMGB1, High mobility group box 1; ICAM, Intercellular adhesion molecule; IFN, Interferon; IL, Interleukin; LFA-1, Leukocyte function-associated antigen-1; IgSF, Immunoglobulin superfamily; LPS, Lipopolysaccharide; MHC, Major histocompatibility complex; MMP, Matrix metalloproteinase; MS, Multiple sclerosis; NCAM, Neural cell adhesion molecule; NE, Norepinephrine; NGF, Nerve growth factor; NO, Nitric oxide; NT, Neurotrophin; PRR, Pattern recognition receptors; RAG, Recombination activating gene; RAGE, Receptor for advanced glycation end products; SCID, Adaptive immunity of severe combined immunodeficiency; SDF, Stromal cell-derived factor; SIRPα, Signal regulatory protein-α; TCR, T cell receptor; TGF-β, Transforming growth factor-β; Th, T helper; TIMP, Tissue inhibitor of metalloproteinase; TLR, Toll-like receptor; TNF, Tumour necrosis factor; Treg, Regulatory T cells; TREM, Triggering receptor expressed on myeloid cells; VIP, Vasoactive intestinal peptide.

## Competing interests

The authors declare that they have competing interest.

## Authors’ contributions

LT prepared the manuscript and the figure. LM and TK revised the manuscript. ZL proofread the manuscript. All authors read and approved the final manuscript.
